# Amoebic liver abscess

**DOI:** 10.1590/0037-8682-0665-2021

**Published:** 2022-02-25

**Authors:** Chee Yik Chang, Anuradha P. Radhakrishnan

**Affiliations:** 1 Hospital Selayang, Medical Department, Selangor, Malaysia.

**FIGURE 1:**
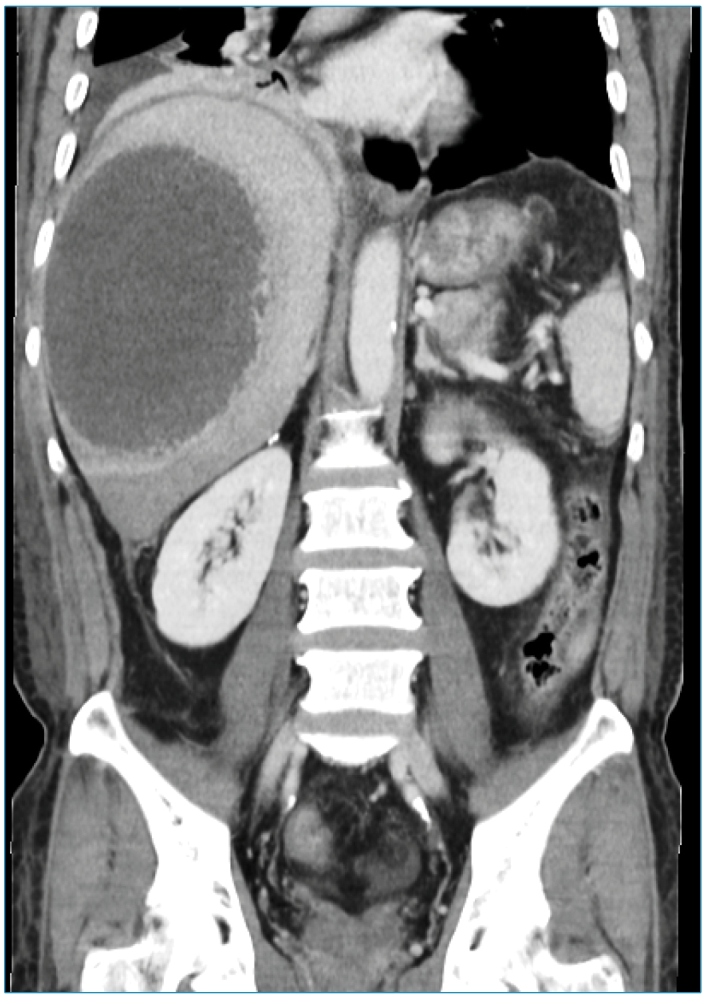
Computed tomography of the abdomen showing a large non-enhancing hypodense liver lesion, suggestive of a liver abscess.

**FIGURE 2:**
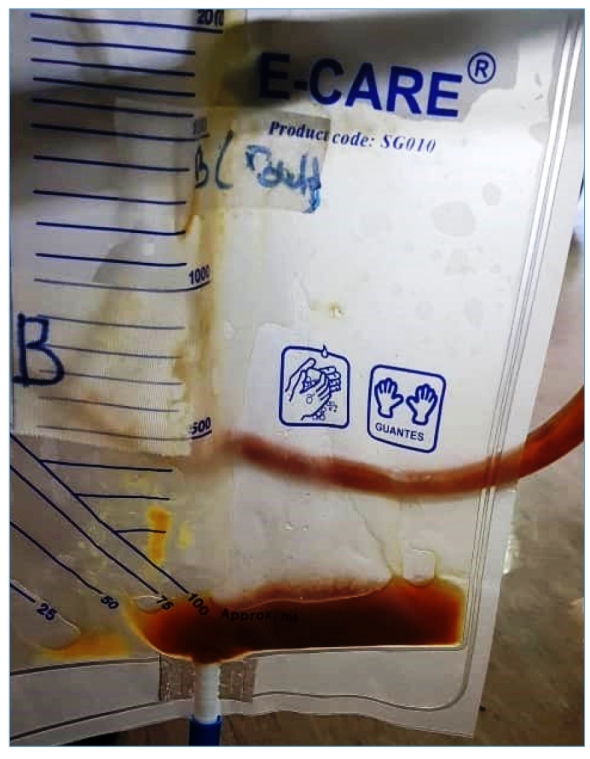
Aspirate from the liver abscess revealed a thick yellow-brown liquid resembling “anchovy paste”.

A 63-year-old Aboriginal fisherman presented with fever and right hypochondriac pain for one week, preceded by non-bloody diarrhea two days prior. Physical examination revealed hepatomegaly, but no jaundice or other signs of chronic liver disease. Computed tomography (CT) of the abdomen revealed a large non-enhancing hypodense lesion at segments V, VI, VII, and VIII measuring 12.5 x 9.8 x 13.8 cm with septations noted within (Figure 1). Overall, the findings indicated a partially liquefied right liver lobe abscess. Percutaneous ultrasound-guided catheter drainage of the liver abscess was performed, and an odorless thick yellow-brown liquid, commonly described as "anchovy paste," was aspirated (Figure 2). Amoebic liver abscess was suspected because of the risk factors, single solitary liver lesion, and "anchovy paste" aspirate. Blood and aspiration cultures were negative, but amoebiasis serology was positive, confirming the diagnosis of amoebic liver abscess. The patient received intravenous metronidazole 500 mg every eight hours for a total of 10 days. A repeat CT scan revealed that the liver abscess had resolved three months later.

Amoebic liver abscess is the most common extra-intestinal manifestation of *Entamoeba histolytica* infection, which is transmitted via the fecal-oral route. Patients with amoebic liver abscess typically present with fever and right upper quadrant pain. Other symptoms include diarrhea, dysentery, and jaundice. Amoebic liver abscesses are more likely to be solitary lesions than multiple lesions, and they are more commonly found in the right lobe of the liver than in the left lobe^1^ ^,^ ^2^. Gross examination of the aspirate may occasionally reveal the probable cause of the liver abscess. Amoebic liver abscess aspirate is odorless, thick, and chocolate brown, and is described as "anchovy paste." On the other hand, pyogenic liver abscess aspirate has a foul odor and is purulent^3^. Amoebic liver abscess is diagnosed with a combination of typical imaging findings and positive amoebiasis serology. Drainage of amoebic liver abscesses is not recommended as a routine treatment unless there is a high risk of abscess rupture, or the patient does not respond to metronidazole-based medical therapy^1^ ^-^ ^3^.
